# Ischemic Heart Disease Mortality and Long-Term Exposure to Source-Related Components of U.S. Fine Particle Air Pollution

**DOI:** 10.1289/ehp.1509777

**Published:** 2015-12-02

**Authors:** George D. Thurston, Richard T. Burnett, Michelle C. Turner, Yuanli Shi, Daniel Krewski, Ramona Lall, Kazuhiko Ito, Michael Jerrett, Susan M. Gapstur, W. Ryan Diver, C. Arden Pope

**Affiliations:** 1 Department of Environmental Medicine, and; 2 Department of Population Health, New York University School of Medicine, Tuxedo, New York, USA; 3 Healthy Environments and Consumer Safety Branch, Health Canada, Ottawa, Ontario, Canada; 4 McLaughlin Centre for Population Health Risk Assessment, University of Ottawa, Ottawa, Ontario, Canada; 5 Department of Environmental Health Sciences, University of California, Berkeley, Berkeley, California, USA; 6 Epidemiology Research Program, American Cancer Society, Atlanta, Georgia, USA; 7 Economics Department, Brigham Young University, Provo, Utah, USA

## Abstract

**Background:**

Fine particulate matter (PM2.5) air pollution exposure has been identified as a global health threat. However, the types and sources of particles most responsible are not yet known.

**Objectives:**

We sought to identify the causal characteristics and sources of air pollution underlying past associations between long-term PM2.5 exposure and ischemic heart disease (IHD) mortality, as established in the American Cancer Society’s Cancer Prevention Study-II cohort.

**Methods:**

Individual risk factor data were evaluated for 445,860 adults in 100 U.S. metropolitan areas followed from 1982 through 2004 for vital status and cause of death. Using Cox proportional hazard models, we estimated IHD mortality hazard ratios (HRs) for PM2.5, trace constituents, and pollution source–associated PM2.5, as derived from air monitoring at central stations throughout the nation during 2000–2005.

**Results:**

Associations with IHD mortality varied by PM2.5 mass constituent and source. A coal combustion PM2.5 IHD HR = 1.05 (95% CI: 1.02, 1.08) per microgram/cubic meter, versus an IHD HR = 1.01 (95% CI: 1.00, 1.02) per microgram/cubic meter PM2.5 mass, indicated a risk roughly five times higher for coal combustion PM2.5 than for PM2.5 mass in general, on a per microgram/cubic meter PM2.5 basis. Diesel traffic–related elemental carbon (EC) soot was also associated with IHD mortality (HR = 1.03; 95% CI: 1.00, 1.06 per 0.26-μg/m3 EC increase). However, PM2.5 from both wind-blown soil and biomass combustion was not associated with IHD mortality.

**Conclusions:**

Long-term PM2.5 exposures from fossil fuel combustion, especially coal burning but also from diesel traffic, were associated with increases in IHD mortality in this nationwide population. Results suggest that PM2.5–mortality associations can vary greatly by source, and that the largest IHD health benefits per microgram/cubic meter from PM2.5 air pollution control may be achieved via reductions of fossil fuel combustion exposures, especially from coal-burning sources.

**Citation:**

Thurston GD, Burnett RT, Turner MC, Shi Y, Krewski D, Lall R, Ito K, Jerrett M, Gapstur SM, Diver WR, Pope CA III. 2016. Ischemic heart disease mortality and long-term exposure to source-related components of U.S. fine particle air pollution. Environ Health Perspect 124:785–794; http://dx.doi.org/10.1289/ehp.1509777

## Introduction

Numerous epidemiologic studies have documented associations between long-term exposure to fine particulate matter mass ≤ 2.5 μm (PM_2.5_) air pollution and increased mortality in urban populations (e.g., Beelen et al. 2014; Brook et al. 2010; Dockery et al. 1993; Eftim et al. 2008; Krewski et al. 2000; Ostro et al. 2010; Ozkaynak and Thurston 1987; Pope et al. 1995, 2002). This association is notably robust in the United States for cardiovascular disease mortality, and especially death from ischemic heart disease (IHD; associated with a reduction of blood supply to the heart, potentially leading to heart attack), as found in prior analyses of the American Cancer Society (ACS) Cancer Prevention Study-II (CPS-II) cohort (Pope et al. 2004). Based on such studies, the U.S. EPA has attributed nearly 90% of the economic valuation of human health benefits derived from the U.S. Clean Air Act to reductions in PM_2.5_ (U.S. EPA 1999), and nearly 1 in 5 U.S. IHD deaths are associated with PM_2.5_ exposure (Fann et al. 2012). Globally, it has been estimated by the World Health Organization (WHO) that roughly 3 million people die each year as a result of outdoor ambient particulate matter air pollution exposures (Lim et al. 2012; WHO 2014), indicating that air pollution is one of the world’s largest single environmental health risks, with PM_2.5_ being estimated to account for some 9.4% [95% confidence interval (CI) 6.6–11.8] of all IHD globally (Evans et al. 2013).

Past research has focused primarily on PM_2.5_ mass concentration, so the types and sources of particles most responsible for these adverse health associations are not known, limiting our ability to address this global cardiovascular disease threat. Thus, the National Academy of Sciences (NAS) and the WHO have placed a high priority on determining which constituents and components of the PM_2.5_ mass are most responsible for these reported health effects (NRC 2001; WHO 2007). As noted by the WHO (2007), this would “facilitate targeted abatement policies and more effective control measures to reduce the burden of disease due to air pollution.”

The present analysis addresses this need by extending the follow-up of one of the largest of the PM_2.5_–mortality cohort studies that has linked individual risk factor and ambient air pollution with vital status data (Krewski et al. 2000; Pope et al. 2002, 2004), while expanding the scope to consider both PM_2.5_ source-specific components and trace element constituent exposures. Contributions of this analysis include *a*) expansion of the follow-up from 16 to 22 years, increasing the number of deaths considered by more than one-third; *b*) substantially expanded PM_2.5_ air pollution exposure data, including new PM_2.5_ composition data; and *c*) consideration of source-apportioned PM_2.5_ mass to investigate which pollution sources are most important to PM_2.5_–IHD mortality associations in this cohort.

## Methods

### Study Population

The study population cohort was drawn from the ACS CPS-II, a prospective mortality study of approximately 1.2 million adults (Garfinkel 1985; Krewski et al. 2009). Participants were enrolled by ACS volunteers in the fall of 1982, and resided in all 50 states, the District of Columbia, and Puerto Rico. Enrollment was restricted to persons at least 30 years of age, in households including someone at least 45 years of age, from whom the ACS obtained informed consent. Participants completed surveys that included questions about age, sex, weight, height, smoking history, alcohol use, occupational exposures, diet, education, marital status, and other characteristics. This secondary analysis of the ACS CPS-II data set was approved by the Ottawa Hospital Research Ethics Board, Canada, and by the Institutional Review Board at the New York University School of Medicine.

Deaths were ascertained through personal inquiries (e.g., direct contact with participants by volunteers) through September 1988, and subsequently via the National Death Index (Calle and Terrell 1993) through 31 December 2004. Mortality from IHD was studied because this was the category found most associated with PM_2.5_ exposure in past analyses of this cohort (e.g., Krewski et al. 2009; Pope et al. 2004). More than 99% of known deaths were assigned a cause using the *International Classification of Diseases, 9th* and *10th Revision* (ICD-9 codes 410–414; ICD-10 codes I20–I25). The analytic cohort included 445,860 participants having questionnaire, known vital status through 2004, and contextual census data, residing in 100 U.S. metropolitan areas within the contiguous United States where the required air pollution data were available, with 34,408 IHD deaths (of a total of 157,572 deaths from all causes) occurring during follow-up. Seventy-six of the metropolitan study areas were included in previously published ACS studies (e.g., Pope et al. 2002, 2004).

### Particulate Matter Source and Constituent Exposure Estimates

**Figure 1:**
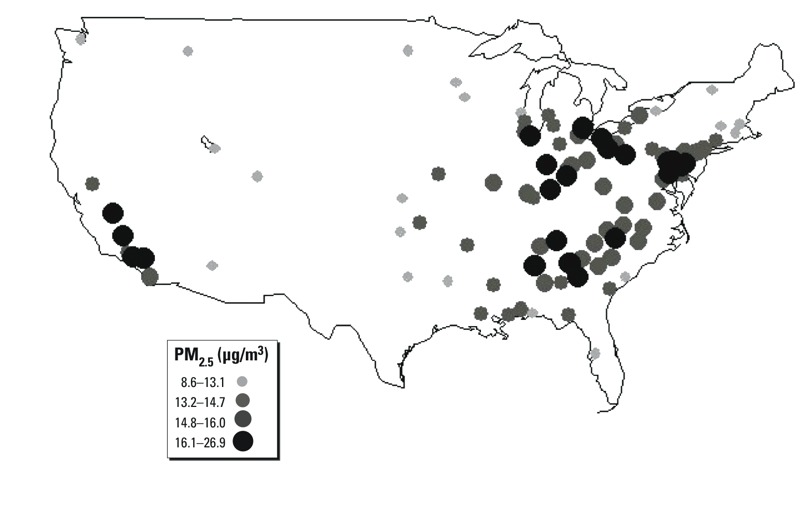
Mean PM_2.5_ mass concentration in each of the 100 metropolitan statistical areas [developed for 2000–2005 from the Health Effects Institute (HEI) Atmospheric and Environmental Research (AER) database (HEI 2007)].

Participants were assigned a metropolitan area of residence based on their enrollment address and three-digit ZIP code area. Complete data were available for 100 metropolitan statistical areas (MSAs) (Figure 1). The mean concentrations of PM_2.5_ mass and trace constituents were compiled for 2000–2005 from the Health Effects Institute (HEI) Atmospheric and Environmental Research (AER) database (HEI 2007), derived from the U.S. EPA Air Quality System that archived Chemical Species Network (CSN) and gaseous criteria pollutant data. The present analysis focuses on further investigations of the PM_2.5_–IHD association, so gaseous air pollutants, evaluated in past analyses (Krewski et al. 2009), are not considered here, except in the derivation of the source factors (Thurston et al. 2011), as discussed below. These PM_2.5_ constituent data were analyzed to derive estimates of source apportioned PM_2.5_ mass exposure concentrations using the absolute principal component analysis (APCA) PM_2.5_ source apportionment method (Thurston and Spengler 1985). Because this process results in orthogonal source components, the source impacts developed have the advantage that they are derived to be as independent of one another as possible. This method involved *a*) a factor analysis of the trace constituents; *b*) identification of source-related factors (based on key tracers in each component); *c*) adjustment of factor scores into absolute scores; and *d*) a regression of the PM_2.5_ mass data on the source-related components, yielding apportionments of PM_2.5_ mass to each source-related factor (Thurston et al. 2011).

These PM_2.5_ mass and speciation data have been previously characterized on a nationwide basis (Bell et al. 2007). Trace elements considered were arsenic (As), calcium (Ca), chlorine (Cl), iron (Fe), lead (Pb), manganese (Mn), nickel (Ni), selenium (Se), vanadium (V), silicon (Si), zinc (Zn), potassium (K), sodium (Na), elemental carbon (EC), organic carbon (OC), and sulfur (S).

In the source apportionment analysis of these trace constituent and mass data (Thurston et al. 2011), the major U.S. PM_2.5_ source categories derived (and their key source-identifier elemental tracers) were metals industry (Pb, Zn); soil particles (Ca, Si); motor vehicle traffic (EC); steel industry (Fe, Mn); coal combustion (As, Se); oil combustion (V, Ni); salt particles (Na, Cl); and biomass burning (K, OC). Although these various tracer elements are individually emitted by multiple sources, when they appear together they have been found to be useful for identifying specific source category impacts (e.g., both As and Se for coal) (Thurston et al. 2011). Sulfur (S) is considered individually here, but it is a general marker for fossil fuel sources that could have different predominant origins in different places (e.g., oil or diesel combustion in California vs. coal combustion in the Eastern United States), so it is not as uniquely associated with any one single source category, and was therefore not included in the source apportionment model (Thurston et al. 2011). Of traffic emissions, EC derives predominantly from diesel vehicles, whereas nitrogen dioxide (NO_2_), also included as a traffic tracer in this source apportionment, is emitted by both diesel- and gasoline-powered vehicles (Schauer et al. 2006). Nationwide spatial plots of the source-related PM_2.5_ contributions to total PM_2.5_ mass (hereafter referred to as source impacts) have been found to support the source-component interpretations in this analysis: Ubiquitous sources, such as traffic and soil, were found to be spread across the nation, while more unique sources were not (e.g., steel and metals processing highest in select industrialized cities, coal highest in the Ohio River Valley region, biomass burning highest in the U.S. Northwest, and residual oil combustion highest in the northeastern United States and major seaport cities) (Thurston et al. 2011). To provide directly comparable results with past nationwide analyses of this cohort (e.g., Krewski et al. 2009; Pope et al. 2002, 2004), individual-site elemental and source apportioned PM_2.5_ exposures were averaged over the concentration values for all monitors in each MSA for the health effects analyses (ranging from one to four PM_2.5_ speciation monitoring stations per MSA) over the period 2000–2005, with a majority (53%) of participants residing in an MSA with multiple sites.

### Statistical Analysis

Standard and multilevel random-effects Cox proportional hazard (RE-CPH) regression models were applied to estimate mortality in relation to each of the individual indices of PM_2.5_ air pollution. The most extensively adjusted models estimated IHD mortality hazard ratios (HRs) for each PM_2.5_ mass, constituent, and source exposure using Cox proportional hazards (CPH) (Fleming and Harrington 1991) models that also included spatial random effects and control for contextual socioeconomic variables. We also evaluated models with random effects but not contextual variables, models with contextual variables but not random effects, and fixed-effects models adjusted for individual-level variables only. The time axis used in the models was survival time from enrollment date. Survival times were censored at time of death or end of follow-up. To facilitate comparisons with past analyses, we modeled individual PM_2.5_ constituents and sources as continuous variables and report HRs for interquartile range (IQR) increases (equal to the difference between the 25th and 75th percentile) in the estimated mean concentration of each pollutant during 2000–2005. We also report HRs per 1-μg/m^3^ increases in each source to facilitate comparisons among sources according to each source’s mortality contribution per unit mass. In addition, we conducted sensitivity analyses of simultaneous CPH models, including both a source-related PM_2.5_ mass and the remaining PM_2.5_ mass (i.e., PM_2.5_-source–related PM_2.5_), for each identified PM_2.5_ source category to estimate the source-specific PM_2.5_ IHD impacts, after adjusting for the impact of all other PM_2.5_. Natural spline plots, showing concentration–response curves for key source impact categories, were also prepared, using the statistical package R. In R, a model was defined using the function ns() of the R “splines” package, in combination with the GAMEPHIT procedure, by including in the model a term the form ns(x, df = k), where df is the number of degrees of freedom (Krewski et al. 2009; R Core Team 2012). All survival models were stratified by 1-year age categories, sex, and race (non-Hispanic white vs. other), assigning each sex–race–age category its own baseline hazard.

A total of 42 variables were included to control for individual characteristics that might confound the air pollution–mortality association. The variables included were individual-level risk factors from the CPS-II enrollment questionnaire (Garfinkel 1985), based upon previous ACS air pollution analyses model specifications (e.g., Krewski et al. 2009), as listed in Table 1. These included active smoking and former smoking (yes/no for each; and cigarettes/day, duration, and initiation < 18 years among active and former smokers, respectively); ever cigar/pipe smoker (yes/no); passive smoke exposure (hours/day); possible workplace exposure to PM (yes/no); occupational dirtiness index (7 categories with an indicator of exposure to an increasingly dirty job or not, plus a missing data category); marital status (married, single, or other); education (< high school, high school, or > high school); body mass index (BMI) and BMI^2^; consumption of beer, wine, and other alcohol (yes, no, or missing, with separate variables for each type of alcohol); quintile of dietary fat consumption; and quintile of combined dietary vegetable, fruit, and fiber consumption. For the occupational dirtiness index, six enrollment question variables were used to record past occupational exposures to asbestos, chemicals/acids/solvents, coal/stone dust, coal tar/pitch/asphalt, diesel engine exhaust, or formaldehyde, and these were then collapsed into one variable for the statistical analyses by identifying a participant as occupationally exposed if a “1” for “yes” appeared for any one of the six variables (Krewski et al. 2000).

**Table 1:** Descriptive statistics of CPS-II cohort characteristics as a function of PM_2.5_ concentration quartile.

Variable	Entire cohort (*n* = 445,860)	PM_2.5_ quartiles (μg/m^3^)			
		8.60–13.35 (*n* = 109,313)	13.36–15.16 (*n* = 109,684)	15.17–16.49 (*n* = 101,775)	16.50–26.93 (*n* = 125,088)
No. of MSAs	100	26	31	23	20
Age (years)	56.6 ± 10.5	57.4 ± 10.6	56.5 ± 10.4	56.3 ± 10.6	56.5 ± 10.4
Male sex (%)	43.6	43.9	43.7	43.5	43.3
Non-Hispanic white race (%)	94.1	96.3	94.5	92.2	93.3
Education
Less than high school	12.5	11.8	13.5	11.7	13.0
High school	31.3	31.3	32.8	29.3	31.6
More than high school	56.2	56.9	53.7	59.0	55.4
Smoking status
Current smokers
% of participants	21.6	20.0	22.1	22.0	22.3
No. of cigs/day	22.1 ± 12.5	22.1 ± 12.6	22.2 ± 12.5	21.9 ± 12.5	22.2 ± 12.6
Duration (year)	33.3 ± 11.1	33.6 ± 11.0	33.2 ± 10.9	33.3 ± 11.2	33.2 ± 11.1
Started smoking < 18 years	41.0	40.2	41.6	40.9	41.2
Former smokers
% of participants	25.3	25.4	25.2	25.8	24.8
No. of cigs/day	21.3 ± 14.9	21.3 ± 14.7	21.6 ± 15.0	21.1 ± 14.9	21.3 ± 14.9
Duration (year)	22.1 ± 12.6	21.9 ± 12.6	22.2 ± 12.5	22.0 ± 12.6	22.2 ± 12.7
Started smoking < 18 years	38.3	37.1	39.2	39.2	37.9
Exposure to smoking (hr/day)	3.2 ± 4.4	2.9 ± 4.3	3.3 ± 4.5	3.3 ± 4.4	3.3 ± 4.5
Ever pipe or cigar smoker only (%)	9.7	9.4	9.8	10.0	9.6
Marital status
Married	83.7	84.9	83.6	83.8	83.7
Single	3.5	3.0	3.5	4.2	3.4
Other	12.8	12.1	12.9	13.0	13.3
Body mass index	25.1 ± 4.1	25.1 ± 4.0	25.3 ± 4.1	25.0 ± 4.1	25.2 ± 4.1
Occupational dirtiness index
Level 0	50.4	50.6	49.5	50.4	51.1
Level 1	13.0	12.6	13.5	13.5	12.6
Level 2	11.2	11.9	10.5	11.6	10.8
Level 3	4.7	4.8	4.9	4.5	4.7
Level 4	6.4	6.7	7.3	6.0	5.7
Level 5	4.3	4.4	4.6	3.8	4.2
Level 6	1.2	1.0	1.2	1.0	1.4
Not able to ascertain	8.8	8.0	8.5	9.2	9.5
Industrial exposures (%)	19.7	19.5	20.2	19.0	19.9
Fat consumption
1st quintile	14.5	13.4	14.7	14.9	14.8
2nd quintile	15.9	15.4	15.9	15.9	16.2
3rd quintile	17.3	17.4	17.1	17.3	17.5
4th quintile	21.1	21.6	21.1	20.7	21.1
5th quintile	31.2	32.2	31.2	31.2	30.4
Vegetable, fruit, fiber consumption
1st quintile	17.1	16.2	18.1	16.5	17.2
2nd quintile	20.1	19.5	20.8	19.8	20.2
3rd quintile	18.9	18.8	18.9	19.2	18.7
4th quintile	22.6	23.0	22.0	22.9	22.5
5th quintile	21.3	22.5	20.2	21.6	21.1
Beer consumption
Yes	22.4	22.4	22.8	22.4	22.0
No	9.5	9.5	9.2	9.4	9.7
Missing	68.1	68.1	68.0	68.2	68.3
Liquor consumption
Yes	26.7	28.2	26.1	27.3	25.5
No	8.8	8.6	8.6	8.6	9.1
Missing	64.5	63.2	65.3	64.1	65.4
Wine consumption
Yes	22.0	21.8	21.7	22.5	21.9
No	8.9	9.1	8.8	8.8	9.1
Missing	69.1	69.1	69.5	68.7	69.0
Ecologic risk factors (MSA Level)
Black (%)	10.5 ± 18.0	5.9 ± 12.3	11.9 ± 19.3	14.3 ± 20.2	10.4 ± 18.1
Hispanic (%)	5.2 ± 10.2	6.2 ± 11.2	4.4 ± 8.4	3.4 ± 5.6	6.6 ± 12.9
High-school education or greater (%)	33.8 ± 12.6	35.7 ± 11.6	31.4 ± 11.5	35.4 ± 13.3	33.0 ± 13.4
Unemployment rate (%)	5.4 ± 3.0	5.3 ± 2.4	5.6 ± 3.1	4.9 ± 3.0	5.6 ± 3.4
Gini coefficient of income inequality	0.39 ± 0.05	0.39 ± 0.05	0.39 ± 0.05	0.39 ± 0.05	0.39 ± 0.04
Annual household income ($1,000s)	36.5 ± 13.9	34.7 ± 12.2	35.9 ± 14.0	39.2 ± 15.4	36.5 ± 13.5
MSA, metropolitan statistical area. Values are mean ± SD or percent. PM_2.5_ mass concentration quintiles computed for 2000–2005 using data matched to MSAs from the Health Effects Institute (HEI) Atmospheric and Environmental Research (AER) database. Characteristics are as collected at baseline using the ACS questionnaire (Krewski et al. 2002). These include active smoking and former smoking (yes/no for each; and cigarettes/day, duration, and initiation < 18 years among active and former smokers, respectively); ever cigar/pipe smoker (yes/no); passive smoke exposure (hr/day); possible workplace exposure to PM (yes/no); occupational dirtiness index (seven categories with an indicator of exposure or not, plus a missing data category); marital status (married, single, or other); education (< high school, high school, or > high school); body mass index (BMI) and BMI^2^; consumption of beer, wine, and other alcohol (yes, no, or missing, with separate variables for each type of alcohol); quintile of dietary fat consumption; and quintile of combined dietary vegetable, fruit, and fiber consumption. Six enrollment question variables were used to record past occupational exposures to asbestos, chemicals/acids/solvents, coal/stone dust, coal tar/pitch/asphalt, diesel engine exhaust, or formaldehyde, and these were then collapsed into one variable for the statistical analyses by identifying a participant as occupationally exposed if a “1” for “yes” appeared for any one of the six variables (Krewski et al. 2009).					

Data on ecologic risk factors representing social and economic variables were obtained from the U.S. Census and other secondary sources at the MSA level (U.S. Census Bureau 1993). Six ecologic covariates were obtained from the 1990 U.S. Census [median household income, percentage of persons > 16 years of age who were unemployed, percentage of adults with a postsecondary education, Gini coefficient of income inequality (ranging from 0 to 1, with 0 indicating an equal distribution of income, and 1 indicating that one person has all the income), and percent black and percent Hispanic population] (U.S. Census Bureau 1993). Models also incorporated an MSA-level random effects adjustment, taking into account residual mortality variation within communities (Krewski et al. 2009).

## Results

Key cohort characteristics are tabulated versus PM_2.5_ quintile in Table 1. This is a nationwide cohort, but considers only adults > 30 years of age at enrollment. It was recruited by volunteers, rather than via random sampling. The average age of the cohort was 56.6 years at enrollment, and the group has more advanced education and a higher percentage of white participants than the general population of the United States. The PM_2.5_ constituent and source apportionment exposure data are summarized in Table 2. These data indicate that traffic-related PM_2.5_ has the highest mean concentration averaged across the national cohort (Thurston et al. 2011), whereas other, more regionalized sources (e.g., coal combustion, oil combustion, biomass burning, and soil) have somewhat lower national average PM_2.5_ concentration contributions.

**Table 2:** Descriptive statistics of PM_2.5_ pollution indices, their units, mean ± SD levels, interquartile range (IQR), and 10th–90th percentile range for participants in 100 MSAs during 2000–2005 (derived per Thurston et al. 2011).

PM metric	Units	Mean ± SD	IQR	10th–90th percentile
As	ng/m^3^	1.4 ± 0.5	0.55	0.9–2.0
Ca	ng/m^3^	61.1 ± 35.1	43.8	29.6–100.4
Cl	ng/m^3^	34.6 ± 38.3	33.5	7.8–69.9
Fe	ng/m^3^	106.6 ± 56.2	47.8	59.1–220.9
Pb	ng/m^3^	5.1 ± 3.3	2.6	2.7–9.3
Mn	ng/m^3^	3.7 ± 5.1	2.1	1.5–6.3
Ni	ng/m^3^	2.0 ± 2.5	1.5	0.7–4.0
Se	ng/m^3^	1.4 ± 0.9	0.8	0.7–2.1
V	ng/m^3^	2.5 ± 2.0	2.6	0.8–6.4
Si	ng/m^3^	104.4 ± 55.1	42.9	61.5–157.6
Zn	ng/m^3^	18.0 ± 14.8	10.1	7.6–31.8
K	ng/m^3^	68.4 ± 18.6	21.9	48.0–93.4
Na	ng/m^3^	71.7 ± 48.2	33.4	35.7–141.2
EC	μg/m^3^	0.8 ± 0.3	0.26	0.5–1.1
OC	μg/m^3^	4.4 ± 0.9	0.98	3.5–5.3
S	μg/m^3^	1.3 ± 0.4	0.53	0.7–1.7
PM_2.5_	μg/m^3^	15.0 ± 2.7	3.01	11.3–17.6
Crustal/soil	μg/m^3^	0.8 ± 0.5	0.56	0.4–1.3
Metals-related	μg/m^3^	0.2 ± 0.2	0.18	0.0–0.3
Traffic-related	μg/m^3^	5.5 ± 2.2	2.40	2.9–7.6
Salt aerosols	μg/m^3^	0.1 ± 0.2	0.10	0.0–0.4
Oil combustion	μg/m^3^	1.1 ± 1.1	1.09	0.3–2.2
Steel industry	μg/m^3^	0.0 ± 0.1	0.00	0.0–0.1
Coal combustion	μg/m^3^	1.1 ± 0.9	0.64	0.3–1.9
Biomass burning	μg/m^3^	1.4 ± 0.7	0.54	0.9–2.0

**Figure 2:**
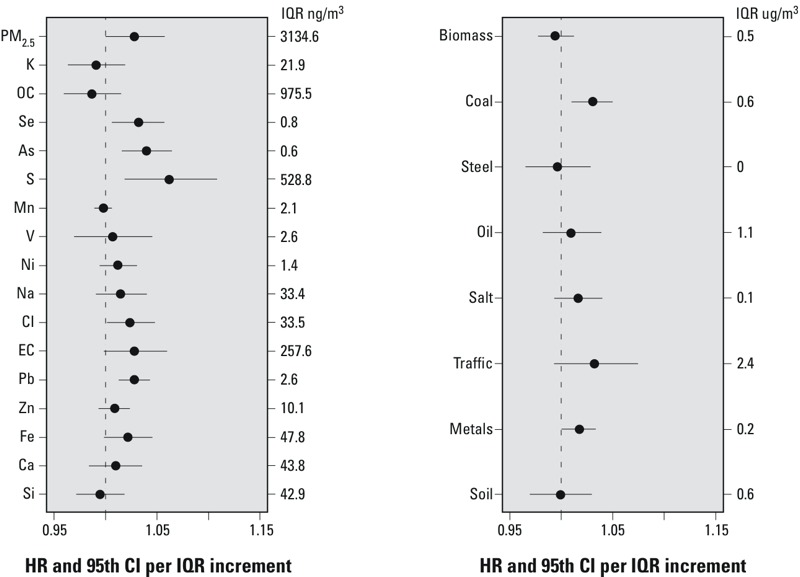
Cox random effects model ischemic disease mortality associations with PM_2.5_ elements and source contributions per interquartile range (IQR). (Steel increment = 0.1 μg/m^3^; steel IQR = 0.0.) Forty-two variables collected at enrollment were included to control for individual characteristics, including active smoking and former smoking; ever cigar/pipe smoker; passive smoke exposure; workplace exposure to PM; occupational dirtiness index; marital status; education; body mass index (BMI) and BMI^2^; consumption of alcohol; dietary fat consumption; and dietary vegetable, fruit, and fiber consumption. Six enrollment question variables were used to record past occupational exposures collapsed into one variable (Krewski et al. 2000, 2009). Six ecologic covariates obtained from the 1990 U.S. Census (median household income, percentage of persons > 16 years of age who were unemployed, percentage of adults with a post-secondary education, Gini coefficient of income inequality, and percent black and percent Hispanic population). These models also incorporated an MSA-level random-effects adjustment.

Figure 2 presents the PM_2.5_–IHD mortality HRs (and 95% CIs) from the RE-CPH model (including individual and contextual variables) for the various PM_2.5_ constituents (i.e., trace elements) and the source components (i.e., PM_2.5_ mass attributed to each source category). The results of all models considered (i.e., CPH with and without random effects, with and without contextual variables) are presented in Table 3. As shown in Figure 2 and Table 3, IHD mortality was statistically significantly associated (*p* < 0.05) with PM_2.5_ mass and a variety of PM_2.5_ elements across most models (e.g., Se, S, As, Cl, Pb, EC, Fe), but consistently not with others (e.g., Si, K, Mn, OC). Estimates based on reduced models (without random effects and/or adjustment for contextual variables) were generally more often statistically significant than the more extensively adjusted model estimates. However, there were some exceptions, for example, the HR central estimate for S based on the fully adjusted model (1.06; 95% CI: 1.02, 1.11) was statistically significant and also larger than the HR adjusted for individual-level variables only (1.02; 95% CI: 1.00, 1.03) (Table 3).

**Table 3:** Trace element and source impact IHD mortality hazard ratios per interquartile range (IQR) for alternative Cox proportional hazard model specifications.

Exposure variable (IQR)	IQR CPH hazard ratio (95% CI)		IQR CPH-RE hazard ratio (95% CI)	
	No random effects, no contextual variables^*a*^	No random effects, with contextual variables^*a*^	With random effects, no contextual variables^*a*^	With random effects and contextual variables^*a*^
PM_2.5_ (3.1347 μg/m^3^)	1.04 (1.03, 1.05)	1.02 (1.01, 1.03)	1.05 (1.02, 1.08)	1.03 (1.00, 1.06)
As (0.00055 μg/m^3^)	1.03 (1.02, 1.04)	1.04 (1.02, 1.05)	1.04 (1.01, 1.06)	1.04 (1.02, 1.06)
Ca (0.04382 μg/m^3^)	1.02 (1.01, 1.03)	1.01 (1.00, 1.03)	1.02 (0.99, 1.05)	1.01 (0.98, 1.03)
Cl (0.03350 μg/m^3^)	1.03 (1.02, 1.04)	1.02 (1.01, 1.03)	1.03 (1.00, 1.05)	1.02 (1.00, 1.05)
Fe (0.04775 μg/m^3^)	1.03 (1.02, 1.04)	1.02 (1.01, 1.03)	1.03 (1.00, 1.05)	1.02 (1.00, 1.05)
Pb (0.00259 μg/m^3^)	1.03 (1.02, 1.04)	1.03 (1.02, 1.04)	1.03 (1.01, 1.04)	1.03 (1.01, 1.04)
Mn (0.00207 μg/m^3^)	1.00 (1.00, 1.01)	1.00 (1.00, 1.01)	1.00 (0.99, 1.01)	1.00 (0.99, 1.01)
Ni (0.00145 μg/m^3^)	1.01 (1.01, 1.02)	1.01 (1.00, 1.02)	1.01 (0.99, 1.03)	1.01 (0.99, 1.03)
Se (0.00081 μg/m^3^)	1.03 (1.02, 1.04)	1.03 (1.01, 1.04)	1.03 (1.01, 1.06)	1.03 (1.01, 1.06)
V (0.00263 μg/m^3^)	1.01 (1.00, 1.03)	1.00 (0.97, 1.01)	1.01 (0.97, 1.05)	1.01 (0.97, 1.05)
Si (0.04288 μg/m^3^)	1.01 (1.00, 1.01)	0.99 (0.98, 1.01)	1.00 (0.98, 1.03)	0.99 (0.97, 1.02)
Zn (0.01013 μg/m^3^)	1.02 (1.01, 1.02)	1.01 (1.01, 1.02)	1.01 (0.99, 1.02)	1.01 (0.99, 1.02)
K (0.02186 μg/m^3^)	1.00 (0.99, 1.02)	1.00 (0.98, 1.01)	1.00 (0.97, 1.03)	0.99 (0.96, 1.02)
Na (0.03337 μg/m^3^)	1.01 (1.01, 1.02)	1.01 (0.99, 1.02)	1.01 (0.99, 1.03)	1.01 (0.99, 1.04)
EC (0.25755 μg/m^3^)	1.04 (1.03, 1.05)	1.03 (1.01, 1.04)	1.03 (1.00, 1.06)	1.03 (1.00, 1.06)
OC (0.97547 μg/m^3^)	1.01 (0.99, 1.02)	0.99 (0.97, 1.00)	1.00 (0.97, 1.03)	0.99 (0.96, 1.01)
S (0.52882 μg/m^3^)	1.02 (1.00, 1.03)	1.05 (1.02, 1.07)	1.03 (0.99, 1.07)	1.06 (1.02, 1.11)
Biomass burning (0.54040 μg/m^3^)	1.00 (0.99, 1.01)	1.00 (0.99, 1.01)	1.00 (0.98, 1.02)	0.99 (0.98, 1.01)
Coal combustion (0.64208 μg/m^3^)	1.02 (1.02, 1.03)	1.02 (1.01, 1.03)	1.03 (1.01, 1.06)	1.03 (1.01, 1.05)
Steel industry^*b*^ (0.1 μg/m^3^)	1.01 (0.99, 1.03)	1.01 (0.99, 1.02)	0.99 (0.95, 1.03)	1.00 (0.97, 1.03)
Oil combustion (1.08574 μg/m^3^)	1.01 (1.00, 1.02)	1.00 (0.99, 1.02)	1.01 (0.98, 1.04)	1.01 (0.98, 1.04)
Salt aerosols (0.10488 μg/m^3^)	1.02 (1.01, 1.02)	1.01 (1.00, 1.02)	1.01 (0.99, 1.03)	1.02 (0.99, 1.04)
Traffic-related (2.39980 μg/m^3^)	1.03 (1.02, 1.04)	1.03 (1.01, 1.05)	1.02 (0.98, 1.05)	1.03 (0.99, 1.07)
Metals-related (0.17518 μg/m^3^)	1.01 (1.00, 1.02)	1.01 (1.01, 1.02)	1.01 (1.00, 1.03)	1.02 (1.00, 1.03)
Crustal/soil (0.56015 μg/m^3^)	1.01 (1.00, 1.02)	0.99 (0.98, 1.01)	1.01 (0.98, 1.05)	1.00 (0.97, 1.03)
^***a***^All models include 42 individual-level variables. Random-effects adjustments are at MSA-level. The six ecological contextual variables were obtained from the 1990 U.S. Census (median household income, percentage of persons > 16 years of age who were unemployed, percentage of adults with a postsecondary education, Gini coefficient of income inequality, and percent black and percent Hispanic population). ^***b***^Steel increment = 0.1; steel IQR = 0.0.				

The source-component results shown in Figure 2 and Table 3 are concordant with the strong associations found for coal combustion’s key tracers (Se and As), with consistently statistically significant IHD mortality–PM_2.5_ source category associations across models being found with coal combustion PM_2.5_ (Table 3). Particulate S, which is predominantly formed from sulfur dioxide emissions from various fossil fuel combustion sources, but especially from coal power plant emissions in the United States during the period of this study (U.S. EPA 2000), is also significant in most models. Metals-related factors and mortality were also statistically significant in some models, though the average exposure is very low across the nation (mean, 0.2 μg/m^3^), consistent with the significant IHD association with Pb, a metal strongly associated with this source factor (Thurston et al. 2011). Coal combustion associated PM_2.5_ yielded an IHD HR of 1.03 (95% CI: 1.01, 1.05, per coal combustion PM_2.5_ IQR = 0.64 μg/m^3^), whereas PM_2.5_ mass yielded an IHD HR of 1.03 (95% CI: 1.00, 1.06 per PM_2.5_ mass IQR = 3.1 μg/m^3^).

When considered on a per 10-μg/m^3^ PM_2.5_ mass basis, the CI of the estimate for PM_2.5_ HR of 1.09 (95% CI: 1.00, 1.20) overlaps prior PM_2.5_–IHD estimates for another subset of this same cohort (e.g., HR = 1.18; 95% CI: 1.14, 1.21 per 10-μg/m^3^ PM_2.5_, from Krewski et al. 2009). When the HRs of the source concentrations (which are much smaller than the total mass) are similarly viewed on a per 1-μg/m^3^ comparable basis, the coal combustion PM_2.5_ had an IHD HR of 1.05 (95% CI: 1.02, 1.08) per 1-μg/m^3^ coal PM_2.5_, versus a 1-μg/m^3^ PM_2.5_ HR of 1.01 (95% CI: 1.00, 1.02). This indicates that the IHD risk estimate for coal PM_2.5_ is roughly five times higher than that for PM_2.5_ mass in general, per microgram/cubic meter. Although the overall traffic-related PM_2.5_ component and the more gasoline engine traffic–related OC were not consistently statistically significantly associated with IHD mortality across the various models, a key tracer of diesel traffic (EC) was positively and statistically significantly associated with IHD in three of the four models considered (*p* < 0.05), and nearly so in the most extensively specified RE-CPH model (HR = 1.03 per 0.3 μg/m^3^ EC; 95% CI: 1.0, 1.06) (Table 3). However, neither wind-blown crustal/soil–related nor biomass combustion–related PM_2.5_ mass was statistically significantly associated with IHD mortality in any model considered.

**Figure 3:**
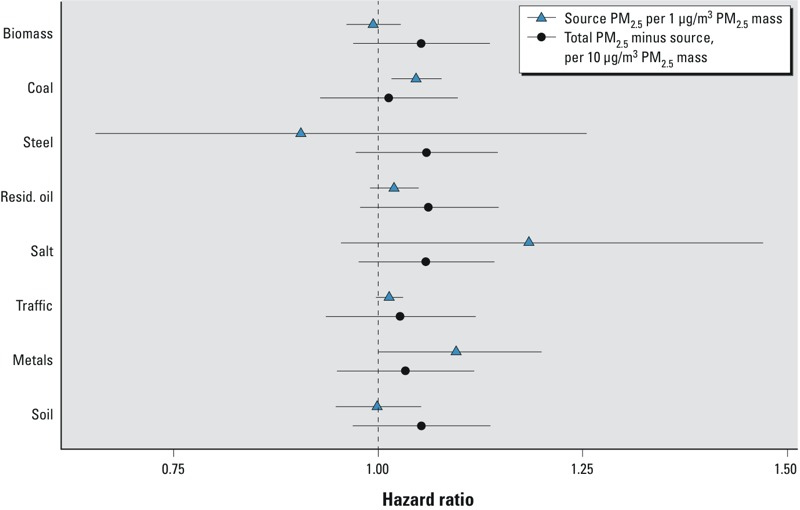
IHD mortality hazard ratios for source-specific PM_2.5_ (HR per μg/m^3^) with other PM_2.5_ mass (i.e., PM_2.5_ mass – source specific mass) (HR per 10 μg/m^3^) simultaneously included in RE-CPH model. Resid., residual. Forty-two variables collected at enrollment were included to control for individual characteristics, including active smoking and former smoking; ever cigar/pipe smoker; passive smoke exposure; workplace exposure to PM; occupational dirtiness index; marital status; education; body mass index (BMI) and BMI^2^; consumption of alcohol; dietary fat consumption; and dietary vegetable, fruit, and fiber consumption. Six enrollment question variables were used to record past occupational exposures collapsed into one variable (Krewski et al. 2000, 2009). Six ecologic covariates obtained from the 1990 U.S. Census (median household income, percentage of persons > 16 years of age who were unemployed, percentage of adults with a post-secondary education, Gini coefficient of income inequality, and percent black and percent Hispanic population). These models also incorporated an MSA-level random effects adjustment. IQRs are listed in Table 1; numeric HRs and CIs are listed in Table 3.

Results of the sensitivity analysis, with both the individual source–related components of PM_2.5_ and the “all other” PM_2.5_ mass for each included simultaneously, are presented in Figure 3. In this analysis, estimates from models of source-related components of PM_2.5_ (presented in Figure 2 and Table 3) were adjusted for a variable representing the remaining PM_2.5_ mass (i.e., after subtracting the mass of each respective source-related component from total PM_2.5_ mass). These results indicate that, even after adjustment for the other PM_2.5_ mass, the coal-related PM_2.5_ HR is the source component least changed, and most clearly seen to be still statistically significant (IHD HR = 1.05; 95% CI: 1.02, 1.08 per 1-μg/m^3^ coal PM_2.5_). In addition, of all the sources considered here, removal of coal-related PM_2.5_ from the PM_2.5_ mass reduces the overall PM_2.5_ mass HR estimate the most. Indeed, once coal-related PM_2.5_ is removed, the “other PM_2.5_” HR drops to nearly 1.0 and is clearly non-significant (IHD HR = 1.01; 95% CI: 0.93, 1.10 per 10-μg/m^3^ PM_2.5_). In these models, diesel traffic–related (IHD HR = 1.01; 95% CI: 1.00, 1.03 per 1-μg/m^3^ PM_2.5_) and metals-related (IHD HR = 1.09; 95% CI: 1.00, 1.20 per 1-μg/m^3^ PM_2.5_) PM_2.5_ are the only other sources that approach statistical significance. Other sources of PM_2.5_ are seen from these results to be less important contributors to the overall PM_2.5_ mass effect because they are clearly nonsignificant, and their removal from PM_2.5_ has a more minor reduction in the PM_2.5_ mass HR estimate.

The correlations of the source-related components, as calculated from the individual trace element observations, were derived to be orthogonal (independent) from one another (Thurston et al. 2011), but averaging the daily values over time and metropolitan areas can introduce intercorrelations among the source impacts (e.g., if two source-specific PM_2.5_ impacts are highest in the same city). The intercorrelations of the various source impacts across the metropolitan areas (not shown) indicate that the highest spatial correlations are found between steel impacts and metals concentrations (*r* = 0.67), which is likely attributable to the fact that both are especially high in Birmingham, Alabama, in this data set (Thurston et al. 2011). The salt- and traffic-related PM_2.5_ components also show a relatively high spatial intercorrelation with each other (*r* = 0.44), which is likely caused by the common use of salt on roads in winter.

**Figure 4:**
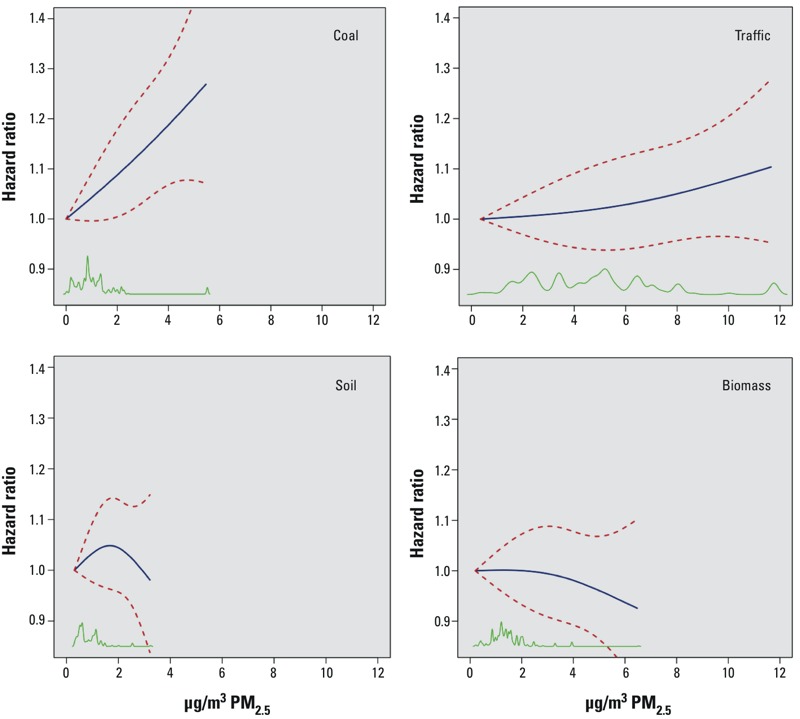
Concentration–response curves (solid blue lines) and 95% CIs (dashed red lines) for source-related PM_2.5_ (μg/m^3^) for coal, traffic, soil, and biomass source contributions to PM_2.5_ mass, in a model specified consistent with the RE-CPH model. Green lines along abscissa indicate data density. Forty-two variables collected at enrollment were included to control for individual characteristics, including active smoking and former smoking; ever cigar/pipe smoker; passive smoke exposure; workplace exposure to PM; occupational dirtiness index; marital status; education; body mass index (BMI) and BMI^2^; consumption of alcohol; dietary fat consumption; and dietary vegetable, fruit, and fiber consumption. Six enrollment question variables were used to record past occupational exposures collapsed into one variable (Krewski et al. 2000, 2009). Six ecologic covariates obtained from the 1990 U.S. Census (median household income, percentage of persons > 16 years of age who were unemployed, percentage of adults with a post-secondary education, Gini coefficient of income inequality, and percent black and percent Hispanic population). All models also incorporated MSA-level random effects.

Natural spline plots of HRs as a function of source-specific mass concentration (Figure 4) are shown for the sources that were most consistently statistically significant across the various models examined (coal and traffic), versus those sources most consistently nonsignificant across the models examined (soil and biomass burning). These are visually seen to be roughly comparable with the Figure 2 RE-CPH HR effect estimates, with coal combustion having the steepest slope (i.e., greatest effect per microgram/cubic meter) presented, and a slope consistent with a 5% increase in IHD risk per 1-μg/m^3^ increase in PM_2.5_ mass concentration from coal combustion. Also, similar to the RE-CPH results, crustal/soil and biomass burning PM_2.5_ are indicated by the CIs provided in these natural spline plots to have nonsignificant associations with IHD mortality.

## Discussion

Although PM_2.5_ pollution from most industrial and fossil fuel combustion categories had estimates of association with IHD mortality > 1.0, coal combustion PM_2.5_ and its key emission tracer elements (i.e., Se and As) were most strongly and robustly associated with IHD mortality across all the various model specifications considered (e.g., of these source and tracer groupings, they are the only ones that were clearly all statistically significant across the four models considered). In addition, particulate S, known to result largely from fossil fuel combustion and especially coal combustion (Zhang et al. 2014), was also found to be associated. Notably, the association between IHD mortality per 1-μg/m^3^ increase in coal combustion PM_2.5_ was larger than the effect estimate for a 1-μg/m^3^ increase in total PM_2.5_ mass. In contrast, IHD mortality was not associated with crustal soil PM_2.5_, biomass PM_2.5_, nor with the elemental tracers associated with these source categories. However, our findings do not rule out effects of sources that were not identified by the source apportionment analysis, or effects of region-specific sources and exposures.

The associations between ambient PM_2.5_ air pollution and IHD mortality in our study cohort are consistent with previous reports, though few studies have estimated associations with long-term PM_2.5_ elemental constituents and/or source-specific PM_2.5_ mass components. A previous cross-sectional study of long-term PM_2.5_ sources and all-cause mortality rates in the United States during 1980 (Ozkaynak and Thurston 1987) similarly reported mortality associations with metropolitan area-wide sulfates and coal combustion-related particle exposures. Previous analyses of the ACS CPS-II cohort (Krewski et al. 2000; Pope et al. 1995, 2002, 2004) and the Harvard Six Cities Study cohort (Dockery et al. 1993) also showed associations between sulfates and both all-cause and cardiopulmonary mortality. Long-term exposures to EC and S were significantly associated with total mortality in a previous analysis of the ACS CPS-II cohort (Smith et al. 2009). A meta-analysis of data from 19 European cohorts indicated that long-term exposure to PM_2.5_ sulfur was associated with natural-cause mortality, and that this association was robust to adjustment for other pollutants and PM_2.5_ mass (Beelen et al. 2015). Sulfate alone, as discussed by Lippmann and Thurston (1996), is an unlikely causal factor for mortality or morbidity from a toxicological perspective, so it may be a contributor to the toxicity of the PM_2.5_ mixture or serve as a marker for a certain type or source of particulate pollution that needs identification. A study of deaths among U.S. veterans (Lipfert et al. 2006) employed data from the U.S. EPA CSN fine particle speciation network, finding mortality associations with nitrates, EC, Ni, V, and traffic density. PM_2.5_ constituents derived from fossil fuel combustion and constituents of crustal origin were more strongly associated than other PM_2.5_ constituents with mortality in a population of 45,000 California teachers (Ostro et al. 2010). A follow-up of this cohort (Ostro et al. 2015) found statistically significant (*p* < 0.05) associations of IHD with PM_2.5_ mass, nitrate, EC, copper, and secondary organics and the sources gas- and diesel-fueled vehicles, meat cooking, and high-sulfur fuel combustion in California. However, individual trace element metals were not associated with CVD mortality in a meta-analysis of 19 European cohorts (Wang et al. 2014). Collectively, these past studies are largely consistent with a finding that the PM_2.5_ association with mortality varies with its elemental composition, but most past studies have not looked at the PM_2.5_ constituent issue from a collective source-specific PM_2.5_ component perspective, which is more readily translatable into air quality policy.

Although effects of short-term (day-to-day) exposures to PM_2.5_ components and sources may differ from effects of long-term exposures, findings from studies of short-term exposures to trace elements and sources have been generally consistent with findings from the present study. For example, Laden et al. (2000) estimated that a 10-μg/m^3^ increase in PM_2.5_ from mobile sources was associated with a 3.4% (95% CI: 1.7, 5.2%) increase in daily mortality in six U.S. cities, and that a 10-μg/m^3^ increase in PM_2.5_ from coal combustion sources was associated with a 1.1% increase (95% CI: 0.3, 2.0%) in mortality. However, daily mortality was not associated with PM_2.5_ crustal soil particles (Laden et al. 2000). The authors concluded that combustion particles in the fine fraction from mobile and coal combustion sources, but not fine crustal particles, were associated with increased daily mortality. Using Bayesian hierarchical modeling, Bell and HEI Health Review Committee (2012) investigated seasonal and temporal variation in PM_2.5_ composition and the risk of total, cardiovascular, and respiratory hospital admissions for persons ≥ 65 years of age in 202 U.S. counties during 1999 through 2005, finding that mortality effect estimates for particulate matter mass were higher in seasons and counties with higher PM_2.5_ Ni content. Ito et al. (2006) applied a Poisson generalized linear model to estimate source-specific PM_2.5_ mass relative risks at lags 0–4 days for total nonaccidental, cardiovascular, and cardiorespiratory mortality adjusting for weather, seasonal/temporal trends, and day-of-week for Washington, DC, during 1988–1997. They found that the secondary sulfate component had the largest (and most consistently statistically significant across investigative teams) percent excess cardiovascular mortality risk estimates, with primary coal-related PM_2.5_ being similarly significantly associated, and risk estimates for traffic-related PM_2.5_, though significant in some cases, were more variable, and soil-related PM showed smaller effect size estimates. These various studies have largely provided results consistent with the findings of this new research, indicating that the mortality risk from PM_2.5_ can vary by mass composition and source.

Overall, in this new research we found that the PM_2.5_ source-component associations with IHD mortality were consistent with results from individual key trace constituents used to identify pollution sources. Model results indicate that, in this data set, long-term exposures to PM_2.5_ from coal combustion-related PM_2.5_ (a source component most correlated with As and Se) and EC (a tracer element from combustion sources, especially diesel-powered motor vehicles in urban areas) were among those most robustly associated with increased risk of IHD mortality across the models considered. The fact that IHD mortality risk was more consistently statistically significantly associated with the EC tracer across the models than was overall traffic-related PM_2.5_ may suggest that the diesel portion of the traffic component increases risk to IHD more than PM_2.5_ from other vehicles. Soil and biomass burning (and their tracers, Si and Ca, and K and OC, respectively) were least often positively significantly associated with IHD mortality across models.

This study has two important strengths. This is the first large nationwide U.S. study to comprehensively examine the characteristics of PM_2.5_ elemental constituents and source-specific components in relation to IHD in a prospective cohort study in which we were able to consider detailed individual-level subject risk factor information. Only recently have the availability of data from the CSN and the development of advanced source apportionment methods made studies of the constituents and sources of PM_2.5_ possible. Second, the large population and number of cities considered, combined with the increased long-term follow-up, provide enhanced power to assess increases in risk related to relatively small changes in PM_2.5_ mass and component exposures.

Despite the strengths of this new analysis, there are several attributes of this study that may have limited its ability to detect all effects. One was the use of central-site monitors as an indicator of individual exposure, which, even though averaged across available sites in each MSA, could potentially introduce nondifferential measurement error across the various sources. Traffic markers such as EC can display significant long-term average concentration spatial variation on scales of 50–500 m within cities (Henderson et al. 2007), whereas elevated pollution sources (e.g., power plants with tall stacks) may be more spatially homogeneous. This could potentially bias the effect estimates of localized sources, such as traffic, toward the null hypothesis of no increase in risk. Another concern might be that not all sources varied as much spatially (e.g., between cities) as others, which may limit the ability of the analysis to identify the impact of those more spatially homogeneous, and less spatially variable, sources, especially the steel industry and salt aerosols (having the smallest overall Standard Deviations in Table 2). However, coal combustion PM_2.5_ exposure had a smaller absolute standard deviation across the United States than two of the other sources (wood burning and traffic), but still had the most significant association, suggesting that this factor was not a major determinant of source significance in this analysis. Spatial confounding may also remain, despite adjustment for contextual variables defined at the MSA level and the use of random effects models to account for uncontrolled regional variation.

Potential misclassification of the cause of death and changes in participant characteristics (such as smoking status) during the follow-up are also sources of potential misclassification. In addition, exposures were classified using PM_2.5_ data from 2000 through 2005, whereas follow-up extended from 1982 through 2004; consequently, there could have been some exposure misclassification if concentrations of PM_2.5_ and PM_2.5_ constituents changed over time, and associations with PM_2.5_ sources in 2000–2005 may actually reflect earlier exposures to these sources of PM_2.5_. Previous analyses of this cohort have shown PM_2.5_ levels at different time periods to be correlated, with lower levels at the end of the follow-up period, but indicating that areas with the highest exposures in the 1980s were still the most highly exposed in 2000 (Pope et al. 2002). Estimated concentrations of many of the trace element constituents (including Si, Se, Zn, V, and Mn) and source components (soil, coal, metals, traffic, and oil) were correlated with estimates for the early 1980s, at the start of cohort follow-up (Thurston et al. 2011), supporting the use of 2000–2005 CSN data to estimate long-term exposures. Although residential mobility is generally relatively low in older adults (Mateyka 2015), exposure misclassification could also have occurred if ACS cohort participants moved to a different area after baseline (Jerrett et al. 2007).

A lack of information on medications (e.g., statins, anti-hypertensives) could also be a potential confounding factor if they are spatially correlated with air pollution exposures, because statins have been suggested as possibly protective against PM_2.5_ health effects (e.g., Schwartz et al. 2005). Because there may have been regional differences in drug-prescribing patterns and adherence, and although adjustment has been made for random effects and for individual- and city-level socioeconomic status indicators, they may not have been adequate to address this potential source of confounding.

These results also raise the causality question as to whether it is biologically plausible that combustion-related particles, and particularly coal combustion PM_2.5_, would have a greater cardiovascular toxicity, per unit mass of exposure, than other particles, especially versus those of biomass or soil origins. There is evidence that oxidant stress is involved in the cardiovascular impacts of PM_2.5_, and it is also known that trace metals, such as those contained in high concentration in fossil fuel combustion–related particles, can cause oxidative stress (Brook et al. 2010), likely contributing to atherosclerosis progression. In addition, New York University’s mouse inhalation studies at five U.S. sites showed substantial variations in aortic plaque progression by geographic region that were consistent with the regional variation in annual IHD mortality in the ACS-II cohort, with both the human and mouse responses being primarily attributable to the coal combustion source category (Lippmann 2014; Lippmann et al. 2013). Moreover, fossil fuel sources, notably coal combustion, are usually higher in sulfur content than biomass particles, resulting in sulfates, a secondary aerosol component that has been associated with increased mortality. The potential interaction of trace metals and sulfur in increasing the toxicity of PM_2.5_ mass has been posited in the recent British Committee on the Medical Effects of Air Pollutants (COMEAP) Report (2009), which states that “Sulphates could play a role in the capacity of PM_2.5_ to drive pulmonary inflammation via either increasing metal mobilisation from PM, and hence increasing the ability to drive oxidative stress leading to inflammation…. Inflammation in the lung could then initiate changes in blood clotting, and/or activation of macrophages in atherosclerotic plaques increasing their instability.”

Although evidence on acute respiratory effects of biomass PM has been well documented (Henderson and Johnson 2012; Martin et al. 2013), evidence of cardiovascular disease impacts by particulate matter from biomass is less certain. Indeed, a recent cohort study of chronic biomass cooking pollution exposures in Bangladesh found a significant increase in respiratory mortality, but not in cardiac deaths (Alam et al. 2012). Although the biological mechanism(s) responsible for the apparently greater long-term cardiovascular toxicity of fossil fuel combustion particles versus others not enriched in transition metals and sulfur (e.g., soil, biomass) are not yet known, there are plausible toxicological pathways consistent with these constituents in fossil fuel PM_2.5_, and especially coal PM_2.5_, being more strongly associated with IHD mortality (Brook et al. 2010). Although our study is a U.S.-based analysis of older adults, these findings have potentially broad policy implications: As the developing world deals with an increasing disease burden from air pollution, reductions in fossil fuel combustion, especially coal combustion, may well be the most efficacious policy approach to reduce air pollution’s global human health burden.

Recent risk assessments have been conducted based on exposure to concentrations of PM_2.5_ mass decomposed into its pollution sources (e.g., Lelieveld et al. 2015). As discussed by Jerrett (2015), these past assessments have generally assumed that the change in risk associated with PM_2.5_ mass concentration is independent of source. Our new study provides evidence that there may, in fact, exist sources where the risk per microgram/cubic meter appears to be either greater than (e.g., coal) or less than (e.g., biomass) the effect estimate derived in past epidemiological studies from the overall mass concentration. Although these new findings are supported by biological plausibility (as discussed above), further research is needed to further test the generalizability of the associations found (e.g., in younger populations).

In summary, we estimated associations between IHD mortality and long-term exposures to elemental constituents and source components of PM_2.5_ in a U.S. cohort of older adults with follow-up from 1982 through 2004. Exposure to fossil fuel combustion–related PM_2.5_ air pollution, especially from coal combustion, but also from diesel traffic–related emissions, was significantly associated with IHD mortality in our study population. This finding, combined with past results and other knowledge of PM_2.5_ constituent toxicity, suggests that the greatest cardiovascular mortality benefits from PM_2.5_ control may be achieved via reductions in fossil fuel PM_2.5_ exposures, especially in PM_2.5_ from coal combustion. This research has implications for both clean air policy and climate change mitigation policy because it provides an indication as to which of the various sources of EC, carbon dioxide, and particulate matter, if reduced, might return the greatest public health benefits.
